# Neoadjuvant endocrine therapy with exemestane followed by response‐guided combination therapy with low‐dose cyclophosphamide in postmenopausal patients with estrogen receptor‐positive breast cancer: A multicenter, open‐label, phase II study

**DOI:** 10.1002/cam4.1600

**Published:** 2018-06-14

**Authors:** Nobuaki Sato, Norikazu Masuda, Takashi Morimoto, Takayuki Ueno, Chizuko Kanbayashi, Koji Kaneko, Hiroyuki Yasojima, Shigehira Saji, Hironobu Sasano, Satoshi Morita, Shinji Ohno, Masakazu Toi

**Affiliations:** ^1^ Department of Breast Oncology Niigata Cancer Center Hospital Niigata Japan; ^2^ Department of Surgery, Breast Oncology National Hospital Organization Osaka National Hospital Osaka Japan; ^3^ Department of Breast Surgery Yao Municipal Hospital Osaka Japan; ^4^ Breast Surgical Oncology Breast Oncology Center Cancer Institute Hospital Tokyo Japan; ^5^ Department of Medical Oncology Fukushima Medical University Fukushima Japan; ^6^ Department of Pathology Tohoku University Miyagi Japan; ^7^ Department of Biomedical Statistics and Bioinformatics Kyoto University Graduate School of Medicine Kyoto Japan; ^8^ Breast Oncology Center Cancer Institute Hospital Tokyo Japan; ^9^ Department of Surgery (Breast Surgery) Graduate School of Medicine Kyoto University Kyoto Japan

**Keywords:** aromatase inhibitors, breast neoplasms, Ki67 index, neoadjuvant therapy, postmenopause, tailored therapy

## Abstract

Patients with estrogen receptor (ER)‐positive breast cancer are less likely to achieve a pathological complete response (pCR) with neoadjuvant chemotherapy. Neoadjuvant endocrine therapy may be more appropriate than neoadjuvant chemotherapy in these hormone‐sensitive patients. Most patients with ER‐positive breast cancer are postmenopausal, and therefore, generally older and less able to tolerate chemotherapy. We aimed to investigate the efficacy and safety of tailored neoadjuvant endocrine and chemoendocrine therapy for postmenopausal breast cancer patients. Untreated patients with primary invasive ER‐positive, HER2‐negative, stage I‐IIIA breast cancer, and Ki67 index ≤30% were enrolled. Patients received exemestane 25 mg/d for 12 weeks. Based on clinical response and change in Ki67 index, assessed at 8‐12 weeks, patients with complete response (CR), partial response (PR) with Ki67 index ≤5% after treatment, or stable disease (SD) with Ki67 index ≤5% before and after treatment were defined as responders. For the subsequent 24 weeks, responders continued exemestane monotherapy (group A), and nonresponders received exemestane 25 mg/d plus cyclophosphamide 50 mg/d (group B). The primary endpoint was clinical response at weeks 24 and 36. A total of 59 patients (median age, 69 years) started initial exemestane monotherapy. After exclusion of three patients who discontinued during this period, 56 remained enrolled to receive subsequent treatment. Clinical response rates (CR and PR) and 95% CI at weeks 24 and 36 were 85% (12/14; 57.2%‐98.2%) and 71% (10/14; 41.9%‐91.6%), respectively, in group A; and 54% (23/42; 38.7%‐70.2%) and 71% (30/42; 55.4%‐84.3%), respectively, in group B. At week 36, no significant difference was found in median Ki67 index between the groups (3.5% and 4.0%). There were no treatment‐related deaths. We found that clinical response comparable to that of responders was achieved in nonresponders after addition of cyclophosphamide to the initial endocrine therapy.

## 1 INTRODUCTION

Neoadjuvant treatment for breast cancer aims to decrease the risk of distal recurrence and to downstage the tumor, thereby allowing less extensive surgery.^1^ It has been shown to be equivalent to adjuvant chemotherapy in terms of disease‐free survival and overall survival.^2^ Prognosis was found to be significantly improved for patients whose operative specimen showed a pathological complete response (pCR), and pCR is now recognized as a factor associated with improved outcomes with neoadjuvant chemotherapy.

Neoadjuvant endocrine therapy may be more appropriate than neoadjuvant chemotherapy in patients with hormone receptor (HR)‐positive breast cancer. These patients are less likely to achieve pCR with neoadjuvant chemotherapy.^3^, ^4^, ^5^ Furthermore, most patients with HR‐positive breast cancer are postmenopausal, and therefore, generally older and less able to tolerate chemotherapy.

Since the introduction of third‐generation aromatase inhibitors (eg anastrozole, exemestane, and letrozole), several studies have been carried out to investigate the effects of neoadjuvant endocrine therapy on HR‐positive breast cancer,^6^, ^7^, ^8^, ^9^, ^10^, ^11^, ^12^ and it has been shown that this therapy may be as effective as neoadjuvant chemotherapy in postmenopausal women with HR‐positive breast cancer.^12^, ^13^ A systemic review and meta‐analysis in 2016 concluded that neoadjuvant endocrine therapy had clinical and radiologic response rates that are similar to those of neoadjuvant chemotherapy, and is associated with less toxicity.^14^ Neoadjuvant chemoendocrine therapy has also been explored; the addition of metronomic cyclophosphamide (ie frequent administration of low‐dose cyclophosphamide) to letrozole resulted in greater reduction of proliferation than with letrozole alone.^15^

However, a marker for the prediction of benefit from neoadjuvant endocrine or chemoendocrine therapy in patients with HR‐positive breast cancer has yet to be established. One candidate predictive marker is Ki67, a nuclear protein expressed by proliferating cells, which is used as a prognostic marker in various types of cancer, including breast cancer.^16^, ^17^, ^18^, ^19^ In several studies, changes in Ki67 index were used to evaluate the effects of neoadjuvant chemotherapy on prognosis in breast cancer patients.^20^, ^21^, ^22^ However, few equivalent studies have been carried out for neoadjuvant endocrine therapy.^6^, ^23^, ^24^ A Ki67‐based scoring system has recently been developed to monitor the response to neoadjuvant endocrine therapy.^25^ We previously showed that 24 weeks’ neoadjuvant endocrine therapy with exemestane provided clinical benefits and significant decreases in Ki67 index in postmenopausal patients with HR‐positive breast cancer.^6^ Notably, there was no progression of tumors with Ki67 index <15% at baseline.

In the present study, we investigated the efficacy and safety of initial neoadjuvant endocrine therapy with exemestane alone in postmenopausal patients with HR‐positive breast cancer. We then classified patients into responders and nonresponders based on clinical response and change in Ki67 index values in response to the initial therapy to evaluate the efficacy and safety of subsequent neoadjuvant therapy (ie continued exemestane monotherapy in responders and exemestane plus cyclophosphamide in nonresponders). The usefulness of Ki67 index as a marker was also examined.

## 2 PATIENTS AND METHODS

### 2.1 Study design and patients

In this multicenter, open‐label, phase II study, patients were enrolled through central registration from eight institutions across Japan. Untreated postmenopausal patients with primary invasive estrogen receptor (ER)‐positive, human epidermal growth factor receptor 2 (HER2)‐negative, stage I‐IIIA (T1c‐T3, N0‐2, M0) breast cancer, Ki67 index ≤30%, and Eastern Cooperative Oncology Group performance status 0 or 1 were eligible. ER‐positive status was confirmed by immunohistochemistry (percentage of positive‐staining cells, ≥1%). HER2‐negative status was confirmed by either immunohistochemistry (score, 1+ or 0) or fluorescence in situ hybridization (HER2/centromeric probe for chromosome 17 ratio, <1.8; or mean number of copies of the HER2 gene, <4 per nucleus).

All patients had adequate hematologic, cardiac, hepatic, and renal function. For each patient, the attending physician had judged neoadjuvant endocrine therapy to be indicated, after consideration of other treatment options, including surgery and neoadjuvant chemotherapy.

The study was carried out in accordance with the Declaration of Helsinki (1975, as revised in 2008) and the Ethical Guidelines for Clinical Research of the Ministry of Health, Labour and Welfare of Japan. The study protocol was reviewed and approved by the institutional review board of each participating institution, and written informed consent was obtained from all patients.

The study is registered with the University Hospital Medical Information Network Clinical Trials Registry (http://www.umin.ac.jp/ctr/index-j.htm), with the unique trial number UMIN000004751. The Japan Breast Cancer Research Group trial number is JBCRG‐11CPA.

### 2.2 Study treatment

**Figure 1:**
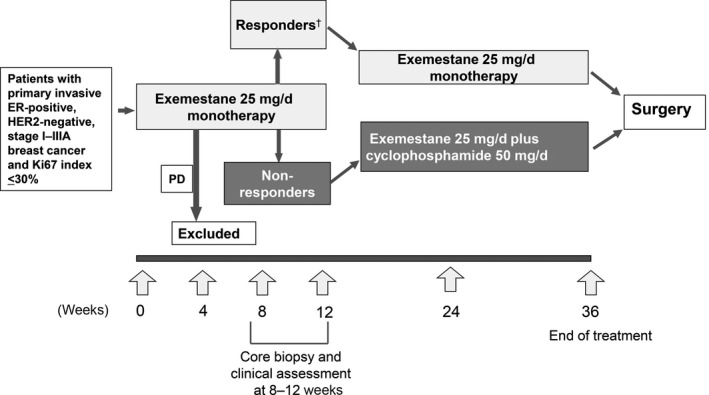
Study design. ^†^Responders were defined as patients with complete response, partial response with Ki67 index <5% after treatment, or stable disease with Ki67 index <5% before and after treatment. PD, progressive disease

Figure 1 shows the study design. Patients first received exemestane alone, at 25 mg/d, administered orally, for 12 weeks. Response to this initial treatment, including effects on Ki67 index values, was assessed at 8‐12 weeks, and patients were registered for treatment for the subsequent 24 weeks as follows: responders continued to receive exemestane monotherapy (group A), whereas nonresponders were switched to combination therapy with exemestane plus cyclophosphamide 50 mg/d, administered orally (group B). The continuation of exemestane therapy for nonresponders was considered potentially beneficial when used in combination with cyclophosphamide therapy at 50 mg/d.

Responders were defined as patients with complete response (CR), those with partial response (PR) and Ki67 index ≤5% after treatment, and those with stable disease (SD) and Ki67 index ≤5% both before and after treatment. Nonresponders were defined as patients with PR and Ki67 index >5% after treatment, and those with SD and Ki67 index >5% either before or after treatment. If Ki67 index could not be determined, patients with CR or PR were classed as responders, and patients with SD as nonresponders.

For patients with progressive disease (PD), the study treatment was discontinued because it was considered to have shown inadequate efficacy in the initial treatment period.

### 2.3 Concomitant and poststudy therapy

Before the initiation of the neoadjuvant endocrine therapy, the status of axillary lymph node metastasis, whether or not sentinel lymph node biopsy was performed, and the estimated extent of mastectomy with least invasion were recorded. After the completion of the study treatment, the status of axillary lymphadenectomy, whether or not sentinel lymph node biopsy was performed, and the actual extent of surgical resection were recorded.

After completion of the study treatment, patients with CR, PR, or SD continued to receive exemestane as postsurgery adjuvant therapy for a total of at least 5 years, including the presurgery treatment period. Also, postsurgery adjuvant chemotherapy and radiation therapy were permitted.

### 2.4 End points

The primary end point was clinical response at weeks 24 and 36. Secondary end points included pathological response; change in tumor size; changes in Ki67 index and preoperative endocrine prognostic index (PEPI) scores derived from the pT stage, pN stage, Ki67 level, and ER status of the surgical specimen;^26^ clinical benefit, assessed as increased rate of conversion from mastectomy to breast‐conserving surgery (BCS); and incidence of adverse events (AEs). AEs were defined as the occurrence or worsening in a patient who received the study treatment of subjective or objective symptoms, or an abnormal change in laboratory test values, that does not necessarily have a causal relationship with the study drug.

### 2.5 Efficacy evaluation

Efficacy was evaluated in accordance with the Response Evaluation Criteria in Solid Tumors, version 1.1. Tumors were assessed at baseline and at 8‐12, 24, and 36 weeks. Additionally to visual inspection and palpation of the breast, tumor size was determined by imaging study (ultrasound; computed tomography, CT; or magnetic resonance imaging, MRI). The choice of imaging study was at the discretion of the attending physician, except for the requirement that CT or MRI had to be carried out additionally to ultrasound for tumors with maximum diameter >4 cm. In cases of multiple tumors, up to five tumors were selected for measurement.

Determination of clinical response was based on comparison of the maximum diameter of tumor(s) with corresponding baseline measurements, or the development of new lesions, as follows: CR, 100% reduction in tumor size; PR, ≥30% reduction in tumor size; PD, ≥20% increase in tumor size, or the development of one or more new lesions; and SD, not CR, PR, or PD. Clinical response rate was defined as the sum of percentages of patients with CR or PR.

Pathological response was determined centrally by the pathology committee (as detailed in the below section), and categorized using the modified criteria previously described by Miller et al^24^ as follows: pCR, when there was no evidence of malignant cell at the original tumor site; pathological partial response (pPR), when histological decrement in cellularity and or increment in fibrosis was detected; or no response, when there was no change.

### 2.6 Procedures of pathological assessment

Tissue samples obtained by core needle biopsy were fixed in 10% formalin (fixation time, 18‐24 hours), and serial 4‐μm paraffin‐embedded sections prepared from selected blocks. For each patient, an unstained slide was sent from each study site to a central laboratory (Tohoku University, Department of Pathology) for immunohistochemical staining to immunolocalize ER, PgR, HER2, and Ki67, as described previously.^27^, ^28^, ^29^

Tissue samples obtained before the start of the study treatment (for diagnostic purposes) were assessed by the pathological committee. The committee determined pathological response after the initial exemestane monotherapy and at the completion of the study treatment using operative specimens. Additionally, tissue samples collected at 8‐12 weeks were used for interim assessment of Ki67 index.

Ki67 index ≤5% and >5% were considered to indicate low and high tumor proliferation, respectively. The cutpoint of 5% was based on the results of a previous study, in which there was no progression of tumors with Ki67 index <15%, and in patients who achieved pPR, median Ki67 index decreased from 10 (range, 0‐55) to 2 (0‐34), that is ≤5%, after 24 weeks of exemestane therapy.^6^ Therefore, we chose Ki67 index ≤5% and favourable clinical response to the initial treatment as the criteria for continuation of exemestane monotherapy.

### 2.7 Safety evaluation

Adverse events were recorded every 4 weeks during the treatment period and graded in accordance with the National Cancer Institute Common Terminology Criteria for Adverse Events, version 4.0 (Japanese Clinical Oncology Group edition).^30^

### 2.8 Statistical analyses

The target sample size was set as 55 patients. This was based partly on the results of calculations using Fleming's single‐stage design for phase II trials (α = 0.05; power, 80%), with a threshold response rate of 54% and an expected response rate of 81% in group A, and a threshold response rate of 26% and an expected response rate of 54% in group B. Also included in the determination of target sample size were response rates achieved in previous studies after 8 weeks of neoadjuvant endocrine therapy with aromatase inhibitors, as well as assumptions about the proportion of patients with Ki67 index ≤5% both before and after exemestane therapy, the proportion with Ki67 index reduced to ≤5% after exemestane therapy, and the number of dropouts.

Tumor response was evaluated by summary statistics; clinical response rate and 95% confidence intervals (CIs) were calculated. The Mann‐Whitney *U*‐test was used to compare continuous variables between groups A and B. McNemar's test was used to compare clinical response rate at weeks 24 and 36. The distribution of AEs was summarized and their incidence rates calculated, stratified by severity (grades 1‐4).

For the evaluation of efficacy, an intent‐to‐treat analysis was carried out; data from all eligible patients were used. The full analysis set was defined as data from all patients who had completed the initial period of treatment with exemestane alone and who started subsequent therapy with either continued exemestane monotherapy or exemestane plus cyclophosphamide. Data from all patients who received at least one dose of the study drug were used in the safety analysis.

Factors associated with classification of patients into responders or nonresponders were identified by univariate and multivariate analyses.

Statistical analyses were performed using IBM SPSS Statistics 23.0 (IBM Corp., Armonk, NY, USA) and R version 3.2.2 (R core team, R Foundation for Statistical Computing, Vienna, Austria).

### 2.9 Follow‐up

Follow‐up of responders and nonresponders is ongoing to investigate the effects on survival in the longer term (disease‐free survival and overall survival) of the tailored approach to neoadjuvant treatment of ER‐positive breast cancer described above. This has been specified in the protocol as a secondary endpoint, and further results will be published in due course.

## 3 RESULTS

### 3.1 Patients

**Figure 2:**
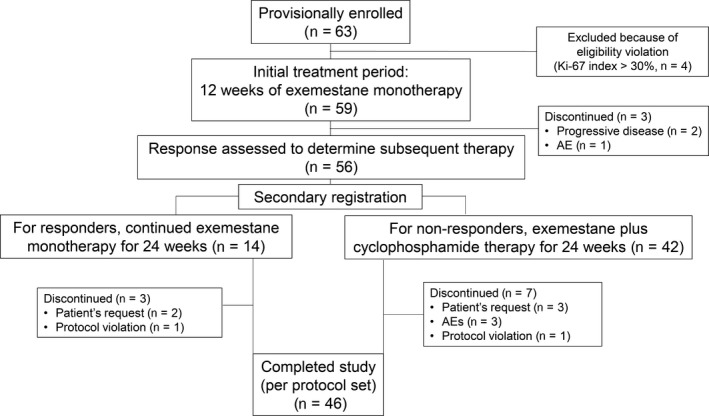
Patient disposition during the study. AE, adverse event

Figure 2 shows patient disposition. A total of 63 patients were provisionally enrolled between January 2011 and July 2015. After exclusion of four patients because of violation of the eligibility criteria (Ki67 index >30%), 59 patients started the initial 12‐week period of treatment with exemestane alone.

Table 1 shows the baseline characteristics of the 59 eligible patients (intent‐to‐treat data set). All had ER‐positive, HER2‐negative breast cancer. None had received prior treatment for breast cancer.

**Table 1:** Baseline patient characteristics (n = 59)^a^

Characteristic	n (%)^b^
Age, y (median and range)	69 (53‐86)
Tumor stage	
T1	8 (14)
T2	49 (83)
T3	2 (3)
Nodal status	
N0	50 (85)
N1	9 (15)
Clinical stage	
I	4 (6)
IIA	49 (83)
IIB	5 (8)
IIIA	1 (2)
PgR status	
Positive	50 (85)
Negative	9 (15)
Ki67 index status	
≤10	32 (54)
>10, ≤20	15 (26)
>20, ≤30	12 (20)

PgR, progesterone receptor.

a Intent‐to‐treat data set. For patients with multiple tumors, the data are for representative lesions only.

b Unless otherwise indicated.

During the initial period of treatment with exemestane alone, three patients discontinued treatment. Of the remaining 56, whose response to the initial treatment was assessed at 8‐12 weeks, 14 were classified as responders (PR and Ki67 index ≤5% after treatment, nine patients; SD and Ki67 index ≤5% both before and after treatment, five patients), and 42 were classified as nonresponders (PR and Ki67 index >5% after treatment, three patients; SD and Ki67 index >5% either before or after treatment, 39 patients). Subsequent treatment, that is, continued exemestane monotherapy (in group A) or combination therapy with exemestane plus cyclophosphamide (in group B), was discontinued by two patients and six patients, respectively. One patient in each group decided not to undergo surgery, therefore, a total of 46 patients completed the study.

### 3.2 Compliance

The rate of compliance, with or without dose reduction, was 93% (13/14 patients) and 83% (35/42 patients) in groups A and B, respectively.

### 3.3 Clinical response at 24 and 36 weeks (primary end point)

Clinical response rates (sum of the percentages of patients with CR or PR) at weeks 24 and 36 were 85% (12/14 patients, 95% CI 57.2%‐98.2%) and 71% (10/14 patients, 95% CI 41.9%‐91.6%), respectively, in group A, and 54% (23/42 patients, 95% CI 38.7%‐70.2%) and 71% (30/42 patients, 95% CI 55.4%‐84.3%), respectively, in group B (Table 2). Clinical response rate at weeks 8‐12 was higher in group A than in group B (*P *= .00). In patients who responded to the initial treatment, clinical response rate remained high with continued exemestane monotherapy. In the nonresponders, clinical response rate improved with subsequent treatment with exemestane plus cyclophosphamide.

**Table 2:** Changes in clinical response rate over the course of the study^a^

Time (weeks)	Group A (continued exemestane monotherapy)		Group B (exemestane plus cyclophosphamide)	
	n (%)	95% CI (%)	n (%)	95% CI (%)
8‐12	9/14 (64)	35.1‐87.2	3/42 (7)	1.5‐19.5
24^b^	12/14 (85)	57.2‐98.2	23/42 (54)	38.7‐70.2
36^b^	10/14 (71)	41.9‐91.6	30/42 (71)	55.4‐84.3

a Clinical response rate defined as the sum of the percentages of patients with complete response or partial response.

b No significant difference was found between clinical response rate at weeks 24 and 36 for either group A or group B (McNemar's test).

### 3.4 Change in tumor size

**Figure 3:**
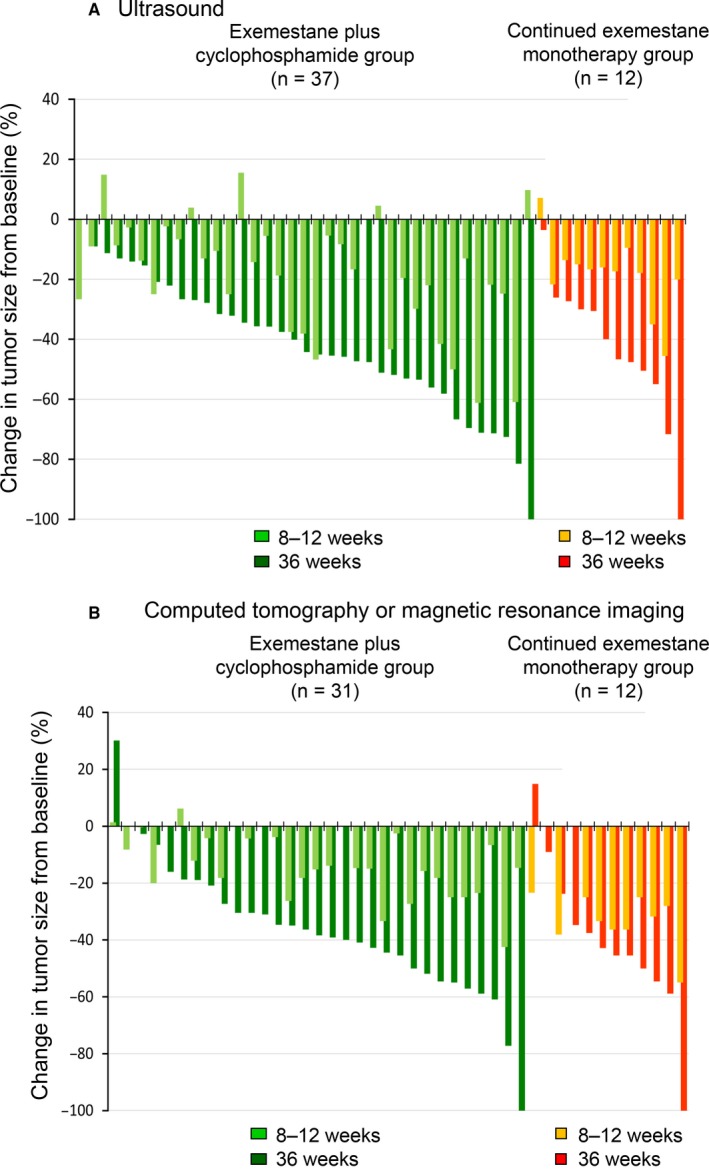
Waterfall plots showing clinical response to exemestane‐based neoadjuvant therapy at 8‐12 weeks and 36 weeks in patients who responded to initial treatment with exemestane alone and who continued to receive monotherapy (group A), and non‐responders, who were switched to exemestane plus cyclophosphamide (group B). Results obtained by (A) ultrasound and (B) computed tomography or magnetic resonance imaging. The horizontal axes indicate paired data from individual patients for whom data were available. The vertical axes show percentage change in tumor size from baseline; positive values indicate tumor progression, and negative values indicate tumor regression

Figure 3 shows changes in tumor size in individual patients at 8‐12 weeks and 36 weeks. Similar results were obtained with ultrasound (Figure 3A) and with CT or MRI (Figure 3B). Regarding the initial tumor regression at 8‐12 weeks, tumor size continued to decrease until 36 weeks in the majority of patients. Most group A patients achieved tumor regression at 8‐12 weeks. Group B patients had a similar degree of tumor regression at 36 weeks.

### 3.5 Change in Ki67 index

**Figure 4:**
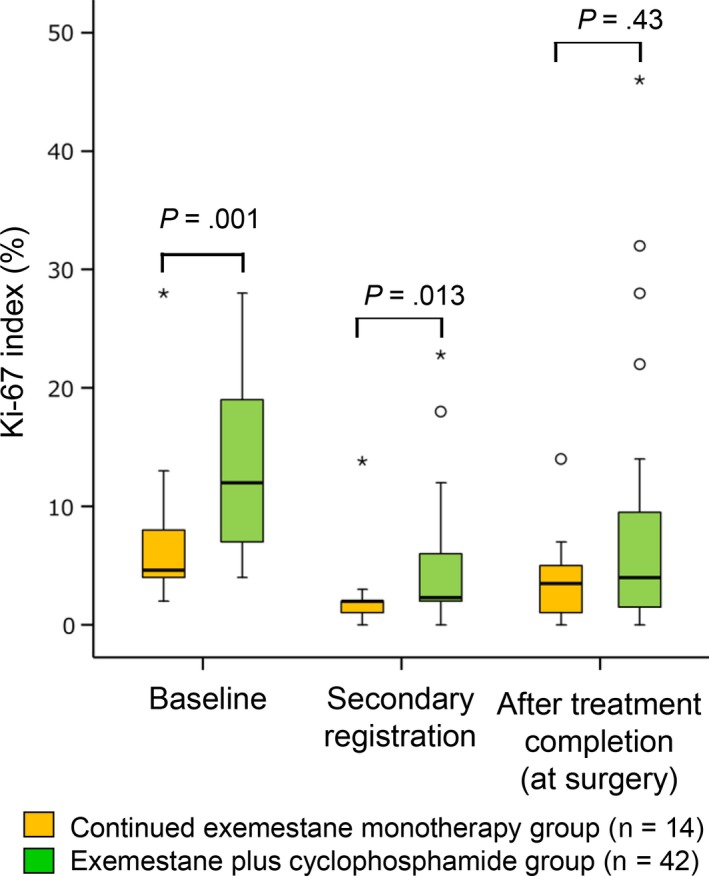
Change in median Ki67 index in patients who responded to initial treatment with exemestane alone and who continued to receive exemestane monotherapy (group A), and non‐responders, who were switched to combination therapy with exemestane plus cyclophosphamide (group B). Data from the full analysis set. * stands for extreme outliers

Figure 4 shows changes in median Ki67 index. Median Ki67 index was significantly lower in responders to the initial treatment with exemestane alone than in nonresponders, both at baseline (4.6% vs 12.0%, *P *= .001) and at secondary registration (8‐12 weeks) (2.0% vs 2.3%, *P *= .013). However, there was no significant difference in median Ki67 index at 36 weeks between group A and group B (3.5% and 4.0%, respectively).

**Figure 5:**
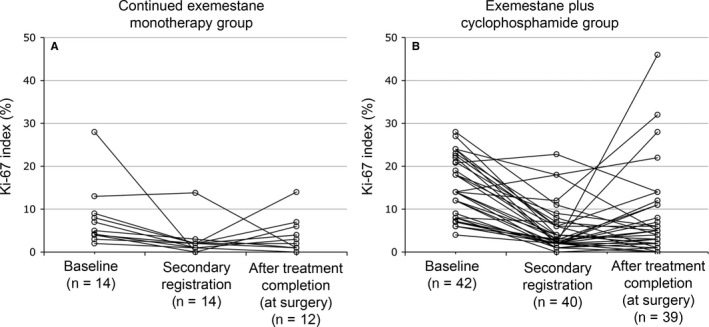
Change in Ki67 index over the course of the study in individual patients in (A) group A (continued exemestane monotherapy) and (B) group B (exemestane plus cyclophosphamide). Baseline data from the full analysis set

Figure 5 shows patterns of change in Ki67 index in individual patients over time. Of the patients who responded to the initial treatment with exemestane alone, Ki67 index decreased during this period in all except one patient, in whom Ki67 index showed a minimal increase. In one patient, the decrease was substantial (about 28%). Ki67 index either increased or decreased during subsequent continued exemestane monotherapy up until surgery but remained <10% in all except one patient. Of the nonresponders to exemestane monotherapy, Ki67 index decreased during the initial treatment period in all except two patients. At the end of exemestane monotherapy, four patients had Ki67 index >10%. Ki67 index decreased further in three of these patients during subsequent exemestane plus cyclophosphamide therapy up until surgery. In four patients, Ki67 index increased to >20% during combination therapy, and in three of these patients the increase was substantial (about 20%‐40%).

### 3.6 Factors associated with response or nonresponse to the initial therapy

The results of univariate and multivariate analyses carried out to identify factors associated with classification of patients into responders or nonresponders, based on clinical response and change in Ki67 index values in response to the initial therapy, are summarized in Table 3. Clinical nodal status N1, ER Allred score 8, and pretreatment Ki67 index <14 were associated with increased likelihood of classification as a responder.

**Table 3:** Factors associated with response or non‐response to the initial therapy

A. Univariate analysis					
Factor	*B*	SE	*P*	OR^a^	95% CI
Age	−0.003	0.05	.95	1.00	0.91‐1.09
≥T2 (ref: T1)	−0.56	1.14	.62	0.57	0.06‐5.34
N1 (ref: N0)	−1.66	0.77	.03	0.19	0.04‐0.85
ER Allred score 8 (ref: <8)	−1.10	0.83	.19	0.33	0.07‐1.70
PgR positive (ref: negative)	0.00	0.88	1.00	1.00	0.18‐5.63
HER2 2+ (ref: negative)	−1.65	0.84	.05	0.19	0.04‐1.00
Ki67 index	0.17	0.07	.02	1.19	1.03‐1.37
Ki67 index ≥14 (ref: <14)	2.37	1.08	.03	10.74	1.29‐89.72

B. Multivariate analysis							
Factor	*B*	SE	*P*	OR^*a*^	95% CI	Model *P*	Cox‐Snell *R* ^2^
Starting model						.007	0.29
Age	0.03	0.06	.58	1.03	0.92‐1.18		
≥T2 (ref: T1)	−2.28	1.71	.12	0.10	0.00‐1.57		
N1 (ref: N0)	−3.84	1.70	.002	0.02	0.00‐0.31		
ER Allred score 8 (ref: <8)	−2.32	1.53	.06	0.10	0.001‐1.08		
PgR positive (ref: negative)	−0.92	1.25	.43	0.40	0.02‐3.39		
HER2 2+ (ref: negative)	−0.59	1.02	.54	0.55	0.08‐4.07		
Ki67 index ≥14 (ref: <14)	1.90	0.98	.03	6.66	1.22‐79.30		
Best‐fit model						.001	0.29
≥T2 (ref: T1)	−2.78	1.78	.06	0.06	0.00‐1.06		
N1 (ref: N0)	−4.41	1.94	.001	0.01	0.00‐0.24		
ER Allred score 8 (ref: <8)	−2.76	1.67	.02	0.06	0.00‐0.72		
Ki67 index ≥14 (ref: <14)	1.89	0.98	.02	6.59	1.25‐68.35		

CI, confidence interval; ER, estrogen receptor; HER2, human epidermal growth factor receptor 2; OR, odds ratio; PgR, progesterone receptor; ref, reference category; SE, standard error.

a Odds ratios for non‐response.

### 3.7 Change in preoperative endocrine prognostic index

The proportion of patients with PEPI score of 0 was similar in group A (3/14 patients, 21%) and group B (10/42 patients, 23%). There was no significant difference in PEPI score between the groups (Table 4).

**Table 4:** Association between treatment group and preoperative endocrine prognostic index (PEPI) score

PEPI score	Group A (continued exemestane monotherapy), n (%)	Group B (exemestane plus cyclophosphamide), n (%)
0	3/14 (21)	10/42 (23)
1‐3	6/14 (62)	21/42 (50)
≥4	4/14 (28)	7/42 (16)
NE	1/14 (7)	4/42 (9)

NE, not evaluable.

### 3.8 Pathological response

In group A, 1 patient had pCR, 12 had pPR, and there were no nonresponders. In group B, 2 patients had pCR, 30 had pPR, and 7 were nonresponders. There was no significant difference in pCR rate between groups A and B (Table 5).

**Table 5:** Pathological response

Pathological response	Group A (continued exemestane monotherapy), n (%)	Group B (exemestane plus cyclophosphamide), n (%)	Both groups, n (%)	*P* (group A vs group B)
Pathological complete response	1 (7)	1 (2)	2 (4)	.5
Pathological partial response	12 (86)	31 (74)	43 (77)	
No response	0 (0)	7 (17)	7 (13)	
Untested	1 (7)	3 (7)	4 (7)	
Total	14 (100)	42 (100)	56 (100)	

### 3.9 Breast‐conserving surgery rate

No increased rate of conversion to BCS was found either in all eligible patients who received exemestane‐based therapy or in the separate groups (data not shown). Based on assessments made before treatment with exemestane, total mastectomy was indicated for 3/14 patients and BCS for 11/14 patients in group A. In group B, total mastectomy was indicated for 10/42 patients and BCS for 32/42 patients. At completion of the study treatment, the proportion of patients who underwent BCS was 71% (10/14 patients) in group A and 69% (29/42 patients) in group B.

### 3.10 Adverse events

There were no treatment‐related deaths. Treatment was discontinued due to elevated liver enzyme in one patient in group B. Other AEs were manageable (Table 6).

**Table 6:** Major adverse events during the treatment period^a^

	Total (n = 56)		Group A (continued exemestane monotherapy) (n = 14)		Group B (exemestane plus cyclophosphamide) (n = 42)	
	Grade ≥3	All grades	Grade ≥3	All grades	Grade ≥3	All grades
All adverse events	7 (13)	30 (54)	1 (7)	3 (21)	6 (14)	27 (64)
Leukopenia	0 (0)	5 (9)	0 (0)	0 (0)	0 (0)	5 (12)
Increased ALT	3 (5)	4 (7)	0 (0)	0 (0)	3 (7)	4 (10)
Increased ALP	0 (0)	3 (5)	0 (0)	0 (0)	0 (0)	3 (7)
Increased AST	0 (0)	3 (5)	0 (0)	0 (0)	0 (0)	3 (7)
Increased γ‐GTP	2 (4)	2 (4)	0 (0)	0 (0)	2 (5)	2 (5)
Hypertension	1 (2)	2 (4)	1 (7)	1 (7)	0 (0)	1 (2)
Arthralgia	1 (2)	2 (4)	0 (0)	1 (7)	1 (2)	1 (2)
Bladder infection	0 (0)	2 (4)	0 (0)	0 (0)	0 (0)	2 (5)
Hepatobiliary disorders (elevated liver enzyme)	0 (0)	1 (2)	0 (0)	1 (7)	0 (0)	0 (0)
Nausea	0 (0)	1 (2)	0 (0)	0 (0)	0 (0)	1 (2)
Diarrhea	0 (0)	1 (2)	0 (0)	0 (0)	0 (0)	1 (2)
Osteoporosis	0 (0)	1 (2)	0 (0)	0 (0)	0 (0)	1 (2)
Gastritis	0 (0)	1 (2)	0 (0)	0 (0)	0 (0)	1 (2)
Hypertriglyceridaemia	0 (0)	1 (2)	0 (0)	0 (0)	0 (0)	1 (2)

ALT, alanine aminotransferase; ALP, alkaline phosphatase; AST, aspartate transaminase; γ‐GTP, γ‐glutamyl transpeptidase.

a Values are expressed as the number (%).

## 4 DISCUSSION

Our main objectives were to investigate, in postmenopausal patients with ER‐positive breast cancer and Ki67 index ≤30%, first, the efficacy and safety of neoadjuvant endocrine therapy with exemestane alone; and second, the efficacy and safety of subsequent neoadjuvant chemoendocrine therapy with exemestane in combination with low‐dose cyclophosphamide in patients who do not respond to exemestane monotherapy.

A predictive biomarker has yet to be established for use in optimizing the neoadjuvant treatment strategy for individual patients with ER‐positive breast cancer. One candidate is Ki67, which is a prognostic marker in breast cancer.^16^ Low and high Ki67 index values indicate low and high tumor proliferation, respectively. In a previous study of neoadjuvant exemestane therapy for primary breast cancer, median Ki67 index decreased from 9 (range, 0‐47) to 2 (range, 0‐37) in patients who achieved PR, and from 8 (range, 1‐68) to 3 (range, 0‐51) in patients with SD.^6^ Additionally, patients with SD had a similar prognosis to those with PR, and posttreatment Ki67 had prognostic value.^31^ Because of our interest in change in Ki67 index as an indicator of treatment efficacy, we chose in the present study to classify patients as responders or nonresponders based primarily on change in Ki67 index values and secondarily on clinical response. Thus, patients with Ki67 index ≤5% both before and after treatment were classified as responders despite having SD, and patients with Ki67 index >5% after treatment were classified as nonresponders despite achieving PR. We believe that Ki67 index value is sufficiently informative to guide clinical decision making for patients with hormone receptor‐positive breast cancer, because triaging breast cancer patients to neoadjuvant chemotherapy on the basis of Ki67 index >10% after neoadjuvant aromatase inhibitor treatment has been shown to be feasible.^32^

The high clinical response rate achieved in patients who responded to initial therapy with exemestane alone was maintained with continued exemestane monotherapy, and the great majority experienced tumor regression at 36 weeks. These findings add to evidence for the efficacy of ≥24 weeks of neoadjuvant exemestane monotherapy.^6^ In nonresponders, clinical response rate improved after the switch to chemoendocrine therapy, and tumor regression was almost universal in this group too. The addition of low‐dose cyclophosphamide may have potentiated the effects of exemestane by inhibition of angiogenesis and vasculogenesis.^15^, ^33^ Regarding safety, exemestane‐based therapy seems to be well tolerated.

Exemestane‐based therapy reduced tumor proliferation. In the first 12 weeks of treatment, substantial decreases in Ki67 index were found in individual patients. Marked decreases in Ki67 expression have also been reported in patients treated with other aromatase inhibitors, including letrozole^24^, ^34^, ^35^ and anastrozole.^23^, ^36^ In patients who were later assessed as having responded to the initial period of exemestane monotherapy, median Ki67 index was significantly lower than in nonresponders both before treatment and at secondary registration (8‐12 weeks). However, median Ki67 index was similar in both groups at 36 weeks. It is plausible that tumors with low proliferation, as indicated by low Ki67 index both before treatment and at secondary registration, are more likely to decrease in size with continued exemestane monotherapy.

PEPI (based on Ki67 index) is a validated tool for prediction of relapse risk in women with early‐stage ER‐positive breast cancer after neoadjuvant endocrine therapy.^26^, ^37^ PEPI scores enable clinicians to tailor subsequent therapy according to risk of relapse, thereby avoiding the use of chemotherapy in low‐risk patients. The American College of Surgeons Oncology Group Z1031A trial is one of the latest to provide support for the assessment of prognosis based on tumor characteristics after neoadjuvant endocrine therapy in postmenopausal women with ER‐positive breast cancer.^32^ Ki67 index was used to decide subsequent treatment after 2‐4 weeks of neoadjuvant therapy with an aromatase inhibitor (anastrozole, exemestane, or letrozole); patients with Ki67 index >10% were switched to neoadjuvant chemotherapy. Risk of relapse over a median of 5.5 years’ follow‐up was significantly lower in patients with a PEPI score of 0 than in those with PEPI >0 (3.7%, 4/109, vs 14.4%, 49/341). The very low risk of relapse (3.6%) in patients with a PEPI score of 0 and treated without chemotherapy suggests that adjuvant endocrine monotherapy may be appropriate in this group. Interestingly, the proportion of patients with PEPI score of 0 in the present study (group A, 21%; group B, 23%) was similar to the proportion in the IMPACT trial (21%) and the POL trial (28%). Further long‐term follow‐up is needed to clarify whether risk of relapse is lower in our patients with a PEPI score of 0, and whether the addition of cyclophosphamide to exemestane therapy affects outcome in these patients.

One aim of neoadjuvant therapy is to increase the likelihood of patients undergoing BCS rather than total mastectomy. However, we found no strong evidence for this in either the responders or the nonresponders, despite the evidence of tumor regression. This contrasts with the findings of previous studies of neoadjuvant therapy using aromatase inhibitors in postmenopausal breast cancer patients.^6^, ^7^, ^8^, ^9^, ^38^

In the present study, at completion of the study treatment, a relatively high proportion of patients underwent BCS (around 70% in each group). Previous studies have shown that neoadjuvant endocrine therapy improves operability and increases the rate of conversion from mastectomy to BCS; however, they included patients with more advanced disease, that is, those who were ineligible for BCS or who had cancer that was inoperable by standard mastectomy. This may explain why no increased rate of conversion to BCS was found in our study, either in the whole group (all eligible patients who received exemestane‐based therapy) or the individual groups.

The finding that neoadjuvant therapy had no significant effect on the rate of conversion from mastectomy to BCS in our study, despite the evidence of tumor regression, raises the question of whether neoadjuvant exemestane therapy is needed in postmenopausal patients with hormone‐sensitive breast cancer. However, we believe that a tailored approach to neoadjuvant treatment of ER‐positive breast cancer is beneficial. Classification of patients into responders and nonresponders, based on clinical response and change in Ki67 index values in response to the initial therapy, enables identification of patients for whom treatment with exemestane alone is more likely to be successful, thereby sparing them the unpleasant effects of chemotherapy.

The results of univariate and multivariate analyses showed clinical nodal status (N1), high ER Allred score and low proliferation to be associated with increased likelihood of response to the initial therapy. Therefore classification of patients into responders or nonresponders, based on clinical response and change in Ki67 index values in response to the initial therapy, seems to reflect biological characteristics rather than tumor volume expressed by clinical nodal status. To help predict long‐term outcomes, PEPI score could be used.

To the best of our knowledge, the present study is the first to investigate the efficacy and safety of combination therapy with exemestane and low‐dose cyclophosphamide in cases of failure to respond to initial treatment with exemestane alone.

The present study has several limitations. First, the study population was limited to 59 eligible patients. Second, although we used a single laboratory for immunohistochemical staining to avoid the problem of variability in Ki67 index measurement across laboratories,^39^ intralaboratory variability remained a possibility. Third, the 83% rate of compliance in group B may have influenced outcomes in this group. Finally, PEPI scores were originally derived from the pT stage, pN stage, Ki67 level, and ER status of the surgical specimen after the initial treatment with aromatase inhibitor alone, and not after aromatase inhibitor plus chemotherapy (ie cyclophosphamide).

## 5 CONCLUSIONS

Our findings provide support for the potential benefit of a tailored approach to neoadjuvant treatment of ER‐positive breast cancer, in which postmenopausal patients with inadequate response (clinical and biologic, ie change in Ki67 index) to initial endocrine therapy are switched to chemoendocrine therapy, thereby maximizing therapeutic effects while minimizing the incidence of AEs associated chemotherapeutic drugs.

Trial number: UMIN000004751 (UMIN Clinical Trials Registry).

## CONFLICTS OF INTEREST

NS received remuneration from Chugai Pharmaceutical, AstraZeneca, Eisai, Pfizer, and Taiho Pharmaceutical. NM received remuneration from Chugai Pharmaceutical, AstraZeneca, and Pfizer, and funding from Chugai Pharmaceutical, Pfizer, Novartis Pharma, AstraZeneca, Eli Lilly, MSD, and Kyowa Hakko Kirin. SS received remuneration from AstraZeneca, Chugai Pharmaceutical, Eisai, Novartis and Pfizer. SM received remuneration from Pfizer outside the submitted work. SO received remuneration from Chugai Pharmaceutical, AstraZeneca, Pfizer, Novartis Pharma, Taiho Pharmaceutical, Sanofi, and Kyowa Hakko Kirin. MT received remuneration from Taiho Pharmaceutical, Novartis Pharma, AstraZeneca, Chugai Pharmaceutical, Genomic Health, has consultant/advisory role at Genomic Health, and received funding from Pfizer. TM, TU, CK, KK, HY, and HS have nothing to disclose.
